# Safety Profile of Anticancer and Immune-Modulating Biotech Drugs Used in a Real World Setting in Campania Region (Italy): BIO-Cam Observational Study

**DOI:** 10.3389/fphar.2017.00607

**Published:** 2017-09-06

**Authors:** Cristina Scavone, Liberata Sportiello, Maria G. Sullo, Carmen Ferrajolo, Rosanna Ruggiero, Maurizio Sessa, Pasquale M. Berrino, Gabriella di Mauro, Liberato Berrino, Francesco Rossi, Concetta Rafaniello, Annalisa Capuano, BIO-Cam Group, G. Valentini

**Affiliations:** The authors would like to thank all the members of the BIO-Cam group who provided patient data for this study: University Hospital of Università degli Studi della Campania “Luigi Vanvitelli” Naples; Hospital SG Moscati—Avellino; Istituto Nazionale Tumori—IRCCS “Fondazione G. Pascale” Naples; Hospital AORN Cardarelli Naples; Hospital G Rummo Benevento; Hospital Sant'Anna e San Sebastiano Caserta; University Hospital Università degli Studi di Napoli Federico II Naples; Fondazione Maugeri Benevento; University Hospital San Giovanni di Dio e Ruggi d'Aragona Salerno; Hospital Ospedale dei Colli Naples; Section of Pharmacology “L. Donatelli”, Department of Experimental Medicine, University of Campania “Luigi Vanvitelli” Naples, Italy

**Keywords:** biotech drugs, safety, real world data, observational study, pharmacovigilance

## Abstract

**Objectives:** To investigate the occurrence of adverse events (AEs) in naïve patients receiving biotech drugs.

**Design:** A prospective observational study.

**Setting:** Onco-hematology, Hepato-gastroenterology, Rheumatology, Dermatology, and Neurology Units in Campania Region (Italy).

**Participants:** 775 patients (53.81% female) with mean age 56.0 (SD 15.2). The mean follow-up/patient was 3.48 (95% confidence interval 3.13–3.84).

**Main outcome measures:** We collected all AEs associated to biotech drugs, including serious infections and malignancies. Serious AEs were defined according to the International Conference on Harmonization of Technical Requirements for Registration of Pharmaceuticals for Human Use, clinical safety data management: definitions and standards for expedited reporting E2A guideline.

**Results:** The majority of the study population was enrolled in Onco-hematology and Rheumatology Units and the most common diagnosis were hematological malignancies, followed by rheumatoid arthritis, colorectal cancer, breast cancer, and psoriatic arthritis. The most commonly prescribed biotech drugs were rituximab, bevacizumab, infliximab, trastuzumab, adalimumab, and cetuximab. Out of 775 patients, 320 experienced at least one AE. Most of patients experienced AEs to cetuximab therapy, rituximab and trastuzumab. Comparing female and male population, our findings highlighted a statistically significant difference in terms of AEs for adalimumab (35.90% vs. 7.41%, *p* < 0.001) and etanercept (27.59% vs. 10.00%, *p* = 0.023). Considering all biotech drugs, we observed a peak for all AEs occurrence at follow-up 91–180 days category. Bevacizumab, brentuximab, rituximab, trastuzumab and cetuximab were more commonly associated to serious adverse events; most of these were possibly related to biotech drugs, according to causality assessment. Three cases of serious infections occurred.

**Conclusions:** The results of our study demonstrated that the majority of AEs were not serious and expected. Few cases of serious infections occurred, while no case of malignancy did. Overall, the safety profile of biotech drugs used in our population was similar to those observed in pivotal trials. Notwithstanding the positive results of our study, some safety concerns still remain unresolved. In order to collect more effectiveness and safety data on biotech drugs, the collection and analysis of real world data should be endorsed as well as the management of post-authorization studies.

## Introduction

In the last thirty years, the global scenario of biotech drugs has grown dramatically. The peculiar feature of biotech products lies in their selective pharmacodynamic activity. According to this characteristic, these products are commonly recognized as “target therapy” (Morrow and Felcone, 2004). Biotech drugs have completely changed the management of several diseases, including cancer and autoimmune diseases such as, psoriasis, rheumatoid arthritis, multiple sclerosis, and inflammatory bowel disease (Cheng and Feldman, 2014). In cancer treatment, the use of biotech agents helped in reducing the common adverse events (AEs) related to standard chemotherapy since they act selectively on cancerous cells, while sparing normal ones, and on specific pathways or proteins strictly related to cancer development (Chan and Hughes, 2015; Pérez-Herrero and Fernández-Medarde, 2015). Given the effectiveness of biotech drugs in cancer therapy, nowadays they represent the backbone of the current anticancer armamentarium. The importance of biotech drugs is well established also in the treatment of autoimmune diseases. Monoclonal antibodies (mAb), such as, infliximab, adalimumab, golimumab, certolizumab pegol, and fusion protein, like etanercept, acting on tumor necrosis factor (TNF) have become mainstay treatment of several autoimmune inflammatory diseases (Curtis and Singh, 2011). Depending on the type and stage of disease, biotech drugs can be used in monotherapy or in add-on to standard treatments (Reang et al., 2006; Hess et al., 2010).

Despite the undeniable advantages offered by these drugs their safety profile is still not completely known, especially for long-term treatments (Day, 2002). One of the major safety concern related to biotech drugs is the development of immunogenicity, which consists in a tendency to trigger an unwanted immune response against self-antigen. Since biotech drugs are engineered molecules, they are more likely to be recognized by the immune system as “invaders,” inducing a harmful production of anti-drug antibodies (ADAs) (Morrow and Felcone, 2004). Generally, ADA production is associated with both reduced clinical efficacy, due to neutralization of therapeutic agent, and increased frequency of major and minor clinical adverse effects, including infusion reactions, mainly related to the development of immune complexes (Morrow and Felcone, 2004; Mellstedt, 2013; van Schouwenburg et al., 2013; Mok et al., 2016; Scavone et al., 2017). Other AEs associated with biotech drugs are infections (Trotta and Valentini, 2005). The antagonism of immune system key components molecules may explain the increased susceptibility of some patients to develop such AE (Ellerin et al., 2003). While premarketing clinical studies did not show an increased risk of serious infections in patients treated with TNF-α inhibitors, epidemiological studies as well as systematic reviews and meta-analysis revealed that patients treated with biotech drugs had an increased risk of bacterial infections than the general population (Mikuls, 2003; Furst, 2010; Bonovas et al., 2016), partly as a result of the underlying disease and partly due to concomitant immunosuppressive drugs.

By inhibiting the activity of the immune system, biotech drugs can also have an important role in cancer immune surveillance with a consequent increase in the frequency of malignancy. To date, only few data are available on this issue and globally suggest that these AEs are very rare (Chakravarty et al., 2005; Bongartz et al., 2006).

Among non-immunological side effects related to biotech therapies, particular attention must be paid to cardiovascular and neurologic AEs. Biotech drugs can induce acute myocardial infarction (Zhang et al., 2016), infusion-related hypertension and myocardial ischemia, cardiomyopathy, and congestive heart failure (Danila et al., 2008; Gasparyan et al., 2012). Trastuzumab, a mAb targeting ErbB2, was linked to cardiotoxicity. It was confirmed that the absence of ErbB2 normal function lead to impossibility for cardiomyocytes to activate survival pathways. Therefore, reactive oxygen species accumulation results in cardiac dysfunction (Onitilo et al., 2014). With regard to neurological complications, biotech drugs can induce multiple sclerosis, optic neuritis, and seizures (Bechtel et al., 2009; Kaltsonoudis et al., 2014). A further neurological AE related to natalizumab, a humanized mAb anti-α4-integrin, is progressive multifocal leukoencephalopathy (PML). It is likely that the drug can induce PML by weakening the central nervous system immune-surveillance, which in turn can enhance the risk of John Cunningham (JC) virus reactivation, the main cause of PML (Boyman et al., 2014).

Therefore, apart from immunological side effects, biotech drugs can also induce AEs target-related and linked to the biological consequences of their action. Cardiotoxicity, neurotoxicity as well as the skin toxicity related to cetuximab and panitumumab, are example of such kind of AEs.

Nowadays, most of our knowledge on biotech drugs' safety profile comes from randomized clinical trials (RCTs). However, due to the strict inclusion criteria, procedures, and ethical issues, RCTs suffer of several limitations, such as, the limited number of enrolled patients, the highly selective population (absence of comorbidities and concomitant treatments), the exclusion of key population patients (elderly, children, and pregnant women), and the short duration. Therefore, data obtained from such studies are not always able to predict AEs in real-world settings. Moreover, in the last thirty years several targeted therapies were approved for the treatment of cancer and autoimmune diseases, but their long-term safety profile have not yet been completely defined. Since clinical evidence derived from pivotal studies could fail to address key safety questions, real world data (RWD; Ruggiero et al., 2012; Iolascon et al., 2013; Cammarota et al., 2014; Ferrajolo et al., 2014; Parretta et al., 2014; Woo, 2014; Menditto et al., 2015; Donati et al., 2016; Giardini et al., 2016; Rafaniello et al., 2016b) should be considered complementary to those obtained from traditional RCTs.

Taking this into account, we carried out a 5-year observational study in naïve patients receiving biotech drugs in several clinical centers in Campania Region with the aim to analyze all AEs, with particular attention to serious infections and malignancies, potentially associated to the aforementioned drugs.

## Methods

### Study design

This was a prospective observational study on the use of biotech drugs, carried out from April 2012 to December 2016, among Onco-hematology (OM), Hepato-gastroenterology (HG), Rheumatology (RT), Dermatology (DM), and Neurology (NE) Units in Campania Region (Italy) on a total of 775 patients. Data come from a large pharmacovigilance network that involved 9 clinical centers (hospital and/or Institute for Treatment and Research) located in different districts of this Region, being representative of the whole Campania Region. The study was approved by the ethic committee of the Coordinating Center of Università degli studi della Campania “L. Vanvitelli”. Patients, identified by the clinicians working within participating centers, were informed about the methods and aims of the project and agreed to participate. A written informed consent was obtained from patients. The study enrolled all patients who received for the first time (naïve patients) a biotech drugs. Pharmacological therapies were chosen only on the basis of clinical judgment and the follow-up visits were planned in accordance with the clinical routine. Follow-up visits consisted of objective examination, blood test, pharmacological treatment revaluation (in term of dose-adjustment, discontinuation, and switch to another drug), and assessment of any AE occurred during the drug therapy.

After patients' enrolment, which coincided with the first biotech administration, the number of follow-up visits per patient varied based on type of disease and related to pharmacological treatment regimen but also on patient's decision. In order to evaluate the occurrence of AEs associated to each biotech drug during the overall study period, we split follow-up period in 6 categories, considering also the injection time (the first biotech administration). The follow-up categories were: “at injection,” 1–30, 31–60, 61–90, 91–180, 181–360, >361 days.

### Demographic and clinical data collection

Standardized monitoring form was supplied to the clinicians to collect demographic and clinical data at the time of the enrolment and at follow-up visits. The enrolment form included the following information: age, sex, clinical diagnosis, type of biotech drug use and exposure time, information on comorbidities; the follow-up form included: type of biotech drug use, in term of dose-adjustment, discontinuation and switch to another drug, AE occurrence (date, seriousness, and suspected drug).

### ADR data collection

When clinicians identified an AE likely associated with biotech drugs, a dedicated section of the standardized monitoring form was filled in. In this section symptoms and signs or diagnosis, date of occurrence, seriousness, and suspected drug were reported. As described in the International Conference on Harmonization of Technical Requirements for Registration of Pharmaceuticals for Human Use, clinical safety data management: definitions and standards for expedited reporting E2A guideline (ICH-E2A, available on line at https://www.ich.org/fileadmin/Public_Web_Site/ICH_Products/Guidelines/Efficacy/E2A/Step4/E2A_Guideline.pdf), a serious AEs corresponds to any untoward medical occurrence that results in death, is life-threatening, requires inpatient hospitalization or prolongation of existing hospitalization, results in persistent or significant disability or incapacity, or results in a congenital anomaly/birth defect or clinically relevant conditions based on clinical judgments.

The reported AEs were coded using preferred terms from Medical Dictionary for Regulatory Activities (MedDRA) and grouped using the System Organ Class (SOCs) classifications of MedDRA. Once ADR was recorded in the dedicated section, according to the current European legislation on pharmacovigilance, clinicians filled in the suspected Adverse Drug Reaction (ADR) reporting form of the Italian Medicine Agency [Agenzia Italiana del Farmaco (AIFA)] and send it to qualified person responsible for pharmacovigilance (QPPV) of their respective health structures. Then, the QPPV recorded the ADR report into a nationwide spontaneous reporting database: the Italian Pharmacovigilance Network (Rete Nazionale di Farmacovigilanza) managed by AIFA. Of note, the decision to report suspected ADRs was exclusively taken by the managing clinicians.

### Data analysis

A descriptive analysis of all AEs reported by the participating centers during the study period was performed. For continuous variables, descriptive statistics (mean, standard deviation, and frequencies) with percentages were calculated. Comparisons using Chi square test were performed (significance level was *p* < 0.05) for categorical variables. Data were analyzed using Microsoft Access and Excel programs.

Since our study was not designed to do a comparison between biotech drugs neither to find a specific association between biotech drugs and AEs, rather to describe all AEs occurring in routine clinical practice, we did not perform any sample size calculation.

We used Naranjo algorithm (Naranjo et al., 1981) in order to establish the strength of relationship between the biotech drug and suspected AEs. All scores ranged between possible and certain reports were considered reasonable for causality. Given the clinical impact of serious AEs and considering that all not serious ones were already expected, we decided to show Naranjo algorithm results exclusively for serious AEs.

## Results

### Clinical and demographic characteristics and biotech drug use

Details on patients' clinical and demographic characteristics are reported in Table 1. Out of 840-screened patients, 775 were enrolled (mean age of 56; standard deviation – *SD* ± 15.2), of whom 53.81% were female. More than 40% of patients had at least one comorbidity, mainly represented by cardiovascular diseases (predominantly hypertension and hypertensive heart disease), diabetes mellitus, and hypercholesterolemia. The mean follow-up/patient was 3.48 (95% Confidence Interval 3.13–3.84).

**Table 1:** Clinical and demographic characteristics of population enrolled.

	**Total**	**Female**	**Male**
N. Patients	775	417	358
Mean age, y (±SD)	56.0 (15.2)	55.4 (14.5)	56.6 (15.9)
Comorbidities	313 (40.39)	171 (41.00)	142 (39.66)
**N. OF FOLLOW-UP**			
No follow-up	263 (33.93)	137 (32.85)	126 (35.20)
1 follow-up	128 (16.52)	72 (17.27)	56 (15.64)
2–5 follow-up	233 (30.06)	128 (30.70)	105 (29.33)
6-10 follow-up	75 (9.68)	41 (9.83)	34 (9.50)
> 10	76 (9.81)	39 (9.35)	37 (10.33)
Mean follow-up/patient (CI 95%)	3.48 (3.13–3.84)		
**CLINIC UNIT**			
Onco-hematology	432 (55.74)	214 (51.32)	218 (60.89)
Rheumatology	219 (28.26)	148 (35.50)	70 (19.55)
Hepato-gastroenterology	77 (9.94)	29 (6.95)	49 (13.69)
Dermatology	33 (4.26)	17 (4.07)	16 (4.47)
Neurology	14 (1.81)	9 (2.16)	5 (1.40)
**DIAGNOSIS (N. %)**			
Hematological malignancies	155 (20.00)	60 (14.39)	95 (26.54)
Rheumatoid arthritis	130 (16.77)	106 (25.42)	24 (6.70)
Colorectal cancer	125 (16.13)	45 (10.79)	80 (22.35)
Breast cancer	88 (11.35)	86 (20.62)	2 (0.56)
Psoriatic arthritis	51 (6.58)	27 (6.47)	24 (6.70)
Crohn disease	41 (5.29)	20 (4.80)	21 (5.87)
Ulcerative colitis	36 (4.65)	8 (1.92)	28 (7.82)
Psoriasis	32 (4.13)	17 (4.08)	15 (4.19)
Spondylo-arthropathy	30 (3.87)	9 (2.16)	21 (5.87)
Head and neck cancer^&^	21 (2.71)	4 (0.96)	17 (4.75)
Lung cancer	19 (2.45)	5 (1.20)	14 (3.91)
Multiple sclerosis	14 (1.81)	9 (2.16)	5 (1.40)
Gastric cancer	12 (1.55)	4 (0.96)	8 (2.23)
Ovarian cancer	9 (1.16)	9 (2.16)	–
Systemic vasculitis	4 (0.52)	2 (0.48)	2 (0.56)
^*^SLE	2 (0.26)	2 (0.48)	–
Osteoporosis	2 (0.26)	2 (0.48)	–
Prostatic cancer	1 (0.13)	–	1 (0.28)
Peritoneal cancer	2 (0.26)	1 (0.24)	1 (0.28)
Missing diagnosis	1 (0.13)	1 (0.24)	–

& *Laryngeal cancer, Brain cancer, and oropharyngeal have been included*.

* *SLE, Systemic lupus erythematosus*.

The majority of the study population was enrolled in OM and RT Units (432 and 219 patients, respectively). In terms of gender distribution, a higher proportion of female patients was enrolled in RT Unit (148 females vs. 70 males), while the opposite situation was seen in HG Unit (29 females vs. 49 males; Table 1).

The most common reported diagnosis were hematological malignancies (N.155 pt; 20%), followed by rheumatoid arthritis (N. 130 pt; 16.77%), colorectal cancer (N. 125 pt; 16.13%), breast cancer (N. 88 pt; 11.35%) and psoriatic arthritis (N. 51 pt; 6.58%). Based on gender distribution, hematological malignancies and colorectal cancers were more common among male patients, while rheumatoid arthritis and breast cancer (as expected) were more frequent in females (Table 1).

**Figure 1:**
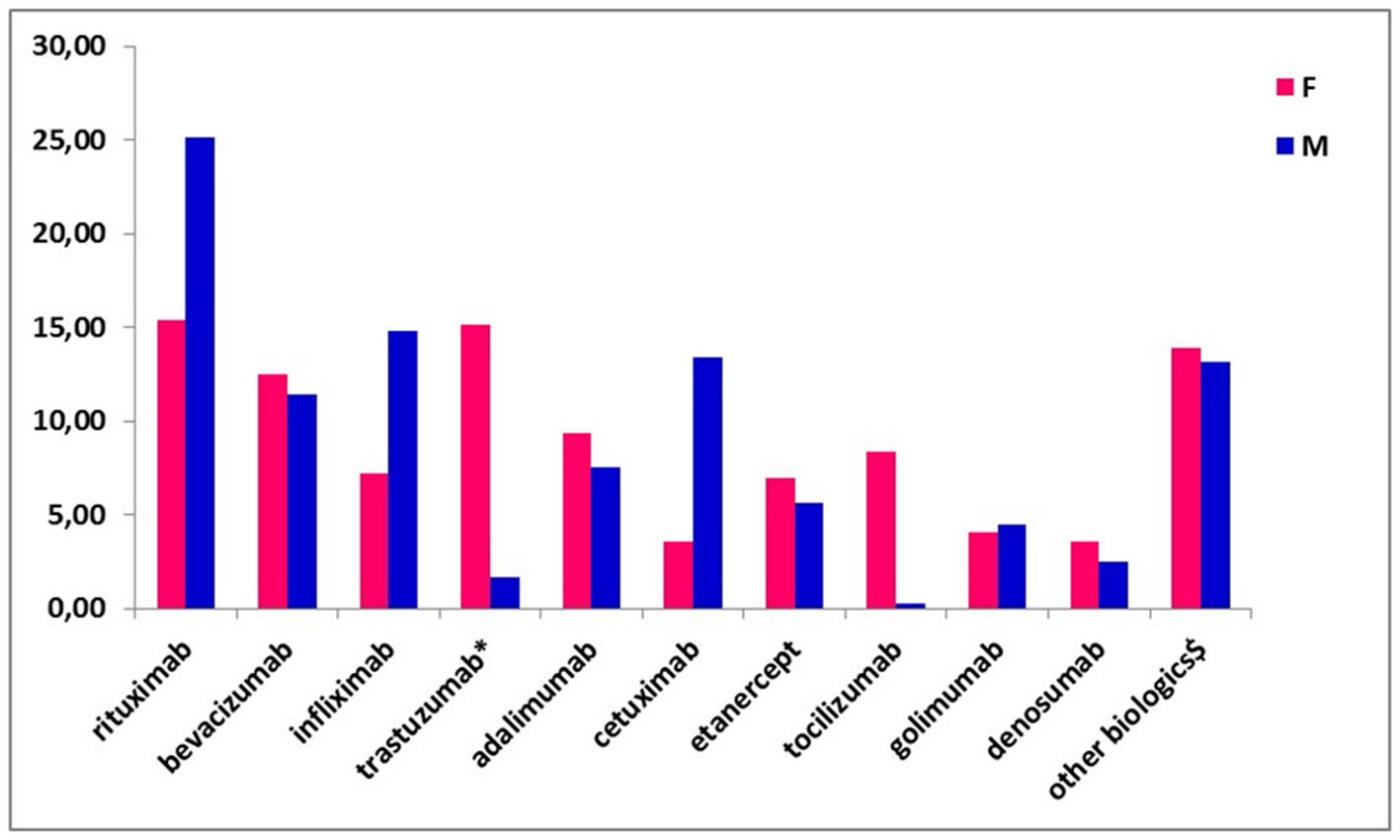
Biotech drug distribution by gender.

The most commonly prescribed biotech drugs at the time of enrolment were rituximab (19.87%), bevacizumab (12.00%), infliximab (10.71%), trastuzumab (including trastuzumab emtansine; 8.90%), adalimumab (8.52%) and cetuximab (8.13%; data not shown). No biosimilar drugs were used in our population. Referring to the drug distribution by gender, trastuzumab and tocilizumab were more commonly used among female patients (15.11 vs. 1.68% and 8.39 vs. 0.28%, respectively), while rituximab and cetuximab were more frequently used among males (25.14 vs. 15.35% and 13.41 vs. 3.60%, respectively; Figure 1).

Some biotech drugs, such as, adalimumab, infliximab and rituximab, were utilized in more than one clinical Unit, thus they have a multiple therapeutic indication.

### Safety

Out of 775 patients, 320 (41.29%) experienced at least one AE (mean of 4.2 AEs per patient, data not shown) with no gender differences (Table 2). Most of patients experienced at least one AE associated to cetuximab (68.25%), followed by rituximab (52.60%) and trastuzumab (47.83%). Adalimumab and etanercept were more frequently associated to AEs in female patients than in male ones (35.90% vs. 7.41%, *p* < 0.001; 27.59% vs. 10.00%, *p* = 0.023; Table 2).

**Table 2:** Distribution of patients with at least 1 adverse event (AE) by gender and type of biotech drug.

	**Total**		**Female**		**Male**		**Chi-square test (*P*-value)**
	**N. users**	**N. with AEs (%)**	**N. users**	**N. with AEs (%)**	**N. users**	**N. with AEs (%)**	
Patients	775	320 (41.29)	417	172 (41.2)	358	148 (41.3)	0.364
**BIOLOGICS**							
Rituximab	154	81 (52.60)	64	37 (57.81)	90	44 (48.89)	0.880
Cetuximab	63	43 (68.25)	15	13 (86.67)	48	30 (62.50)	0.433
Bevacizumab	93	40 (43.01)	52	22 (42.31)	41	18 (43.90)	0.277
Infliximab	83	37 (44.58)	30	14 (46.67)	53	23 (43.40)	0.556
Trastuzumab^*^	69	33 (47.83)	63	30 (47.62)	6	3 (50.00)	0.224
Adalimumab	66	16 (24.24)	39	14 (35.90)	27	2 (7.41)	< 0.001
Golimumab	33	4 (12.12)	17	3 (17.65)	16	1 (6.25)	0.063
Denosumab	24	1 (4.17)	15	–	9	1 (11.11)	–
Etanercept	49	10 (20.41)	29	8 (27.59)	20	2 (10.00)	0.023
Tocilizumab	36	8 (22.22)	35	8 (22.86)	1	–	–
Other biologics^$^	105	47 (34.31)	58	23 (25.00)	47	24 (53.33)	< 0.001

* *Including trastuzumab emtansine*.

$ *Including: abatacept, panitumumab, certolizumab pegol, natalizumab, ustekinumab, pertuzumab, aflibercept, brentuximab vedotin, belimumab, anakinra, brentuximab vedotin, eculizumab, ramucirumab, romiplostin, eculizumab*.

#### AEs distribution by follow-up and SOC

**Figure 2:**
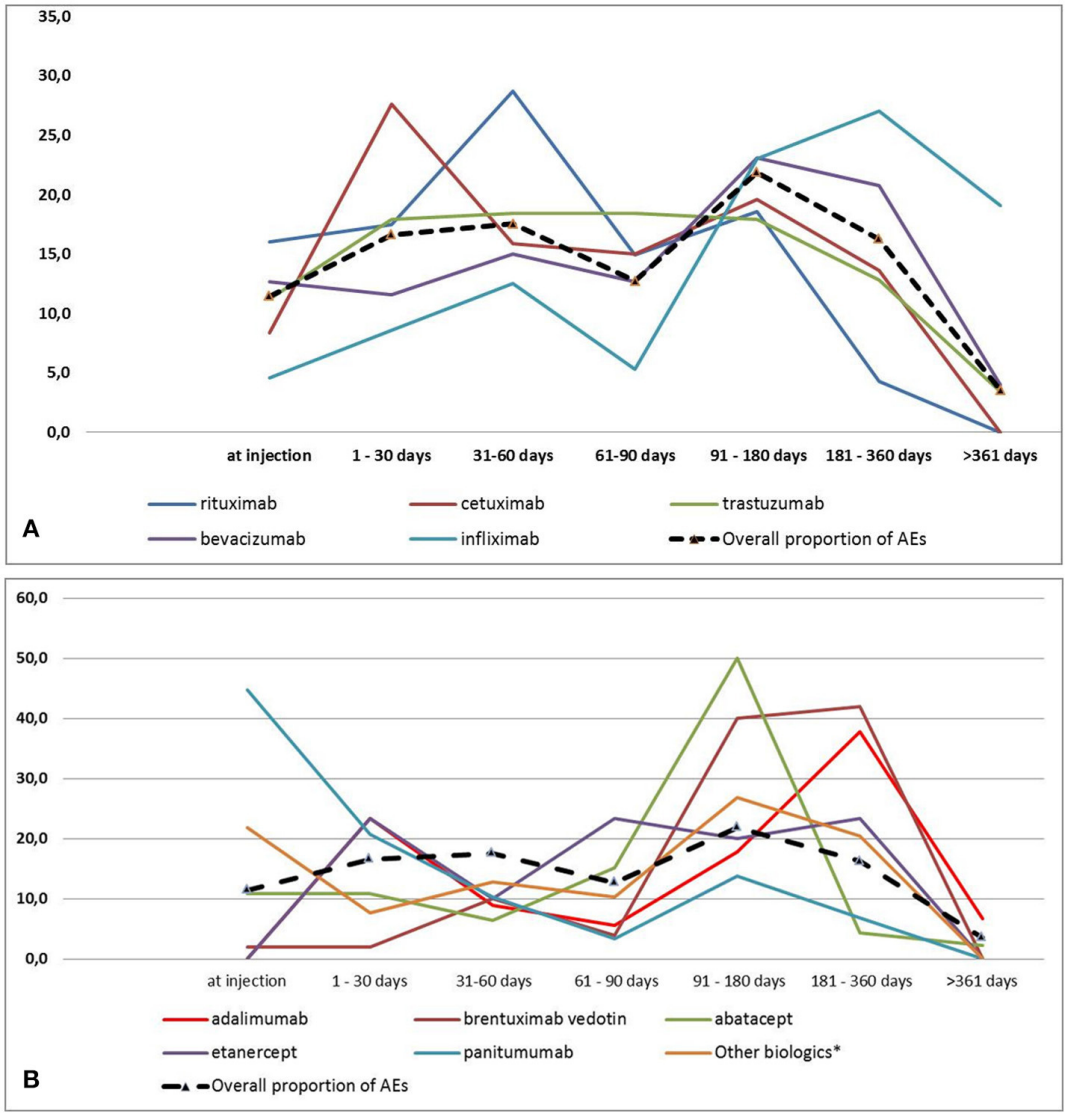
**(A,B)** Adverse events distribution by biotech drugs and follow-up categories.

As shown in Figures 2A,B, considering all biotech drugs, we observed a peak for all AEs occurrence at follow-up 91–180 days category. Cetuximab, rituximab and panitumumab were the ones with an earlier occurrence of AEs (follow-up 1–30, 31–60, and at injection, respectively). AEs related to adalimumab occurred more frequently at 1–30 and 181–360 days. On the contrary bevacizumab, infliximab, and brentuximab vedotin have been reported as suspected drug for delayed AEs with the higher proportion at follow-up 91–180 and 181–360 days. Trastuzumab and etanercept were characterized by a stable frequency of AEs over the study period (Figures 2A,B). At injection, apart from panitumumab which was used in a very low proportion of patients, rituximab was the biotech drug most commonly related to the occurrence of AEs. Finally, biotech drugs related to AEs occurrence at follow-up >361 days were trastuzumab, bevacizumab, infliximab, adalimumab, and abatacept (data not shown).

In terms of SOCs, nervous system disorders, blood and lymphatic system disorders, musculoskeletal and connective tissue disorders, eye disorders, general disorders and administration site conditions and infections were the most commonly identified at 91–180 days category (Supplementary Table 1). The SOCs with an earlier occurrence were skin and subcutaneous tissue, respiratory and vascular disorders with the higher frequency at follow-up 1–30 days). Gastrointestinal disorders were more common at follow-up 31–60 days (Supplementary Table 1).

#### AEs distribution by clinical units

Depending on the therapeutic indication for which each biotech drug was used, a different incidence of AE was observed. For example, bevacizumab was associated to the occurrence of AEs in 58.6% of colorectal cancer patients, 20% of breast cancer patients, 40% of subjects lung cancer diagnosed and 35.7% of those affected by other solid cancers. Overall, the majority of AEs related to bevacizumab was not serious, except when it was used in patients with breast cancer. Similar differences were also observed for cetuximab (Table 3A). Interesting differences were also noted among patients enrolled in other Units. For example, adalimumab was associated to the occurrence of AEs in 36.4% of Crohn disease patients, 50.0% of psoriasis patients, 7.7% of rheumatoid arthritis subjects, 16.7% of psoriatic arthritis patients, and 30% of other rheumatic diseases patients. Most of AEs related to adalimumab was not serious (Table 3B). Finally, although no differences were found in the incidence of AEs related to infliximab, this drug induced more serious AEs in ulcerative colitis patients compared to Crohn disease ones (19.0 vs. 8.1%; Table 3B).

**Table 3A:** Most utilized biotech drugs in OM Unit: occurrence and seriousness of adverse event.

	**Onco-hematology Unit 432 enrolled patients**					
**Diagnosis N. pt (% on 432)**	**Hematological malignancies 155 (35.6)**	**Colorectal cancer 125 (28.9)**	**Breast cancer 88 (20.4)**	**Lung cancer 19 (4.4)**	**Gastric cancer 12 (2.8)**	**Other solid cancers^£^34 (8.1)**
Biologic drug (N. of users, % of patients with AEs, N. of PT reported)	Rituximab (143, 53.8%, 337)	Bevacizumab (58, 58.6%, 133)	Trastuzumab (58, 45.0%, 141)	Denosumab (12, 8.3%, 1)	Trastuzumab (8, 75%, 36)	Bevacizumab (14, 35.7%, 25)
	Brentuximab (8, 87.5%, 50)	Cetuximab (42, 78.6%, 188)	Bevacizumab (15, 20.0%, 3)	Bevacizumab (5, 40%, 11)	Cetuximab (2, 50%, 1)	Cetuximab (17, 47.1%, 18)
	Eculizumab (3, 66.7%, 9)	Panitumumab (19, 73.7%, 20)		(Cetuximab 2, 100%, 8)		
Serious AE by biologic drug (%)	Rituximab (12.2)	Cetuximab (9.0)	Trastuzumab (11.3)	Denosumab (0)	Trastuzumab (13.9)	Bevacizumab (28.0)
	Brentuximab (50.0) Eculizumab (0)	bevacizumab (31.1) Panitumumab (40.0)	Bevacizumab (66.7)	bevacizumab (0) Cetuximab (37.5)	Cetuximab (0)	Cetuximab (38.9)

**Table 3B:** Most utilized biotech drugs in HG, DM, RT, and NE Units: occurrence and seriousness of adverse event.

	**Hepato-gastroenterology Unit N. enrolled patients 77**		**Dermatology Unit N. enrolled patients 32**	**Rheumatology Unit N. enrolled patients 219**			**Neurology Unit N. enrolled patients 14**
Diagnosis N. pt (% on population enrolled in each Clinic Unit)	Crohn disease 41 (53.2)	Ulcerative colitis 36 (46.7)	Psoriasis 32 (100)	Rheumatoid arthritis 130 (59.4)	Psoriatic arthritis 51 (23.3%)	Other^#^ 38 (17.4)	Multiple sclerosis 14 (100%)
Biologic drug (N. of users, % patient with AEs, N. of AEs reported)	Infliximab (30, 56.7%, 124) Adalimumab (11, 36.4%, 30)	Infliximab (33, 45.5%, 42) Adalimumab (2, 100%, 2)	Etanercept (13, 15.4%, 4) Adalimumab (12, 50.0%, 14) Ustekinumab (6, 16.7%, 2)	Tocilizumab (36, 22.2%, 23) Abatacept (20, 40.0%, 44) Etanercept (17, 12.5%, 15) Golimumab (12, 23.1%, 13) Adalimumab (13, 7.7%, 3) Certolizumab pegol (12, 25%, 8) Infliximab (10, 10.0%, 3)	Adalimumab (18, 16.7%, 26) Etanercept (12, 33.3%, 11) Golimumab (9, 11.1%, 1)	Adalimumab (10, 30%, 16) Rituximab (4, 25%, 5) infliximab (3, 33.3%, 2)	Natalizumab (14, 42.9%, 9)
Serious AE by biologic drug (%)	Infliximab (8.1) Adalimumab (0.0)	Infliximab (19.0) Adalimumab (50.0)	Etanercept (0.0) Adalimumab (14.3) Ustekinumab (0.0)	Tocilizumab (34.8) Abatacept (9.1) Etanercept (13.3) Golimumab (15.4) Adalimumab (66.7) Certolizumab pegol (25.0) Infliximab (0.0)	Adalimumab (0.0) Etanercept (9.1) Golimumab (0.0)	Adalimumab (0.0) Rituximab (40.0) Infliximab (100.0)	Natalizumab (33.3)

£ *Other solid cancer includes: head-neck cancer (including oropharyngeal, laryngeal, brain cancers), ovarian cancer, prostatic cancer, peritoneal cancer*.

# *Other: Systemic lupus erythematosus, osteoporosis, systemic vasculitis, spondyloarthritis*.

Analyzing the single AE/biotech drug association reported more than once during the study period among Clinical Units, some differences were highlighted (Table 4). For example, adalimumab was more frequently associated to infusion reactions, upper respiratory tract infections (URTI) and insomnia in patients enrolled in RT unit, generalized pain, myalgia, and infusion reactions in HG patients, and skin eruption in DM patients. Similarly, infliximab was associated to generalized edema and bone or joint pain in RT patients, while seemed to induce more commonly headache, dizziness, and tachycardia in HG patients. Rituximab was associated to paresthesia, nausea and asthenia in OM patients, and to purpura in RT patients.

**Table 4:** AE/biotech drug association reported more than once during the study period by Clinical Units.

	**Onco-hematology**	**Rheumatology**	**Hepato-gastroenterology**	**Dermatology**	**Neurology**
Abatacept	–	Insomnia			
Adalimumab	–	Infusion reactions, URTI, insomnia	Generalized pain, myalgia, Infusion reactions	Skin eruption	
Bevacizumab	Paresthesia, neutropenia, asthenia				
Brentuximab	Paresthesia, neuropathy, diarrhea, headache, cough				
Certolizumab pegol	–	Flu-like syndrome	–	–	–
Cetuximab	Rash, diarrhea, skin fissures				
Eculizumab	Myalgia, constipation				
Etanercept	–	Myalgia, peripheral edema		Hypertension	
Golimumab	–	URTI	–	–	–
Infliximab	–	Generalized edema, bone or joint pain	Headache, dizziness, tachycardia	–	–
Natalizumab	–	–	–	–	Headache
Panitumumab	Rash, skin toxicity	–			
Rituximab	Paresthesia, nausea, asthenia	Purpura	–	–	–
Tocilizumab	–	Itch, abdominal pain, dyspnea, leukopenia, rash	–	–	–
Trastuzumab	Asthenia, neutropenia, diarrhea	–	–	–	–

*URTI, upper respiratory tract infection*.

#### Serious AEs

As reported in Table 5, 140 serious AEs occurred. These were more commonly associated to bevacizumab, brentuximab (including brentuximab vedotin), rituximab, trastuzumab and cetuximab. Specifically, bevacizumab was associated to 32 serious AEs (Table 5), mainly represented by hematological toxicity and peripheral neuropathy; one case of infection was identified (bronchitis) (data not shown). Brentuximab was associated to 24 serious AEs (Table 5), represented by peripheral neuropathy; no serious infections occurred in patients treated with brentuximab (data not shown). Twenty serious AEs occurred in patients treated with rituximab (Table 5). Rituximab-related serious AEs were largely represented by hematological depression and infusion reaction; 2 cases of infections (herpes zoster) were observed (data not shown). Trastuzumab was associated to 16 serious AEs (Table 5). Hematological depression, bleeding–related AEs, and peripheral neuropathy were the most commonly reported AEs; no case of infection was related to trastuzumab therapy (data not shown). Lastly, cetuximab was associated to 14 serious AEs (Table 5), mainly represented by hematological depression, skin and gastrointestinal disorders (data not shown). Applying Naranjo algorithm, causality assessment resulted possible for the majority of serious AEs. Finally, no cases of AEs related to malignancies occurred.

**Table 5:** Serious AEs by biotech drug and causality assessment.

**Biotech drug/(total n. of serious AEs)**	**Therapeutic indications**	**N. serious AEs**	**Causality assessment**
Bevacizumab/(32)	• Colorectal cancer • Breast cancer • Other solid cancer	• 27 • 2 • 3	• 26 possible; 1 probable • 2 possible • 3 possible
Brentuximab^*^/(24)	• Hematological malignancies	• 24	• 24 possible
Rituximab/(20)	• Hematological malignancies • Other rheumatic dis.	• 19 • 1	• 16 possible; 3 probable • 1 possible
Trastuzumab/(16)	• Breast cancer • Gastric cancer	• 13 • 3	• 13 possible • 3 possible
Cetuximab/(14)	• Colorectal cancer • Lung cancer	• 12 • 2	• 12 possible • 2 possible
Infliximab/(11)	• Crohn disease • Ulcerative colitis • Other rheumatic dis.	• 6 • 4 • 1	• 5 possible; 1 probable • 4 possible • 1 possible
Panitumumab/(5)	• Colorectal cancer	• 5	• 5 possible
Tocilizumab/(5)	• Rheumatoid arthritis	• 5	• 5 possible
Abatacept/(4)	• Rheumatoid arthritis	• 4	• 4 possible
Adalimumab/(3)	• Ulcerative colitis • Psoriasis	• 1 • 2	• 1 possible • 2 possible
golimumab/(2)	• Rheumatoid arthritis	• 2	• 2 possible
Natalizumab/(2)	• Multiple sclerosis	• 2	• 2 possible
Certolizumab/(1)	• Rheumatoid arthritis	• 1	• 1 possible
Etanercept/(1)	• Rheumatoid arthritis	• 1	• 1 possible

* *Including brentuximab vedotin*.

### Treatment discontinuation

Fifty patients discontinued the treatment with the biotech drug. Most of these cases were reported for abatacept, bevacizumab, etanercept, and infliximab. Progression disease, drug therapeutic failure and other AEs occurrence represented the main reasons reported of discontinuation (Supplementary Table 2).

## Discussion

In the present study we evaluated the safety profile of anticancer and immune-modulating biotech drugs in a real world setting. Our findings demonstrated that in daily clinical practice such drugs showed a safety profile similar to what observed in RCT, being in general well tolerated.

In line with literature and epidemiological data, our study population was mainly enrolled in OM and RT Units and more than half of study population was affected by hematological malignancies, rheumatoid arthritis and colorectal cancer (Chiu and Weisenburger, 2003; Murphy et al., 2011; Nakajima et al., 2016)^1^^,^^2^. In Campania Region, as well as throughout Italy, the dispensation of biotech drugs, which are indicated for the treatment of both cancer and autoimmune diseases, is heavily regulated and for many of them restricted to the hospital settings.

According to our findings, gender differences in biotech drugs use is mainly related to a different prevalence of specific diseases in female and male patients (Curtis and Singh, 2011)^3^^,^^4^.

As we reported, we found a statistically significant difference in terms of adalimumab- and etanercept-related AEs by gender. Apart from the different prevalence of use of such drugs in male and female population, it is known that female patients have a 1.5- to 1.7-fold greater risk of developing an AE, compared with male ones. Gender-related differences, such as, the ones related to pharmacokinetic (lower lean body mass, reduced hepatic clearance, different mechanism of conjugation, absorption, protein binding, and renal elimination), immunological and hormonal factors could explain this difference (Rademaker, 2001).

### AEs observed with the different biotech drugs

#### Cetuximab

In line with our findings, literature data suggest that skin reactions and gastrointestinal disorders are the most commonly AEs in cetuximab-treated patients, especially in the early stage of treatment (Fakih and Vincent, 2010). Also data from BOND pivotal trial revealed that skin reactions occurred in about 80% of patients within the first 3 weeks after the start of cetuximab therapy and that gastrointestinal AEs were more common among patients who received cetuximab plus irinotecan (Cunningham et al., 2004).

#### Rituximab

Data on rituximab-related AEs are consistent with a literature review, which demonstrated that gastrointestinal AEs usually occur within the first 77 days after the first dose (Kasi et al., 2012). Moreover, apart from gastrointestinal AEs, rituximab can also induce, as already shown (Mohrbacher, 2005), infusion reactions, which can include symptoms such as, asthenia and paresthesia, consistently with what we observed. Rituximab is also associated to the occurrence of skin reactions (Giezen et al., 2012) and cutaneous vasculitis, which usually starts with palpable purpura (Baldo, 2013). Finally, in our population 2 cases of herpes zoster infections were observed. According to data from a pivotal phase III clinical trial, hematological toxicity and infections, including herpes simplex and herpes zoster, can be observed during rituximab treatment (McLaughlin et al., 1998). However, such AEs could be a direct complication of lymphoma and its pharmacological treatments (Gea-Banacloche, 2010).

#### Adalimumab

According to our findings, AEs at injection seems to be very rare (Benucci et al., 2009). Moreover, literature data revealed that adalimumab used in both rheumatology and gastroenterology settings can induce musculoskeletal disorders (Hinojosa et al., 2008; Huang et al., 2009) and URTI (Papp et al., 2012), while data related to the incidence of infections are still controversial (Keyser, 2011). Data from a phase III pivotal trial revealed that the most common AEs related to adalimumab were rash, infusion reactions (including injection site reaction), and pruritus and that no statistically significant difference was detected in the rates of serious AEs between adalimumab- and placebo-treated patients (van de Putte et al., 2004).

#### Infliximab

Most of infliximab-induced AEs, which occurred at both 91–180 and 181–360 days categories, could be related to infusion reactions, which could appear with symptoms, such as, bone and joint pain, myalgia, tachycardia, malaise, and generalized edema (Cheifetz et al., 2003; Steenholdt et al., 2012). The late onset of such AEs could also be explained by the typical infliximab therapeutic schedule, which requires, after the induction stage, the administration of the drug every 8 weeks (Fakih and Vincent, 2010). However, considering that rheumatic disease could itself induce the occurrence of significant pain, erosive joint destruction, and loss of joint function bone, the role of the disease cannot be excluded (Scanzello et al., 2006). Data from pivotal RCTs revealed that infliximab could induce the occurrence of infections, mainly respiratory and urinary, tuberculosis reactivation, usually within 2 months after first infusion, and infusion-related AEs, which are very common and occurred within 1–2 h after the infusion (Keane et al., 2001; Siddiqui and Scott, 2005).

#### Bevacizumab

Consistently with our findings, Smith et al. reported a median time to onset of grade 3–5 AEs equal to 5 months (Smith et al., 2011). While hematologic toxicities can frequently occur during bevacizumab treatment (Schutz et al., 2011), peripheral neuropathies are not so commonly associated to bevacizumab (Grisold et al., 2012). Considering that this drug is used in add-on to standard chemotherapy (i.e., 5-FU, oxaliplatin, irinotecan^5^), in the recommended combinations FOLFOX, FOLFIRI, IFL, XELOX, the role of concomitant agents on the occurrence of such AEs cannot be excluded (Kelly and Goldberg, 2005; Botrel et al., 2016). Regarding to the differences highlighted between colorectal and breast cancer patients, according to Kobayashi and Huang, colorectal cancer patients have higher IL-6 serum level, which in turn could lead to delayed hypersensitivity AEs (Kobayashi et al., 2013; Huang et al., 2014). Lastly, in terms of infections, bevacizumab was associated with a single case of serious bronchitis. Although infections represent expected AEs for the majority of mAb, the real causal relationship between biotech drugs and infection cannot be simply established due to the underlying diseases which, together with concomitant therapies, could themselves cause immunosuppression, leading to infection occurrence (Salvana and Salata, 2009). Data from a bevacizumab pivotal trial demonstrated that gastrointestinal and hematological AEs were common among patients who received this drug, although no difference in the incidence of AEs leading to hospitalization or to treatment discontinuation was detected (Hurwitz et al., 2004).

#### Trastuzumab

Consistently with our findings, which demonstrated that the most common trastuzumab-related AEs were not serious, further studies confirmed that long term use of trastuzumab therapy is safe and well tolerated (Yeo et al., 2015), although the risk of neutropenia could be increased (Tripathy et al., 2004) along with gastrointestinal AEs and asthenia (Balduzzi et al., 2014). In a pivotal phase III trial, the addition of trastuzumab to chemotherapy in women with HER2 overexpressing metastatic breast cancer was associated to the occurrence of serious AEs, which included cardiac dysfunction, asthenia, leukopenia, dyspnea, and infusion reaction (Leonardi et al., 2010).

#### Etanercept

Etanercept showed a constant trend in AEs occurrence in all follow-up categories, apart from at injection time. This finding could be explained by the time of onset of injection site AEs, which frequently appear within 24–48 h after administration (Huang et al., 2009). According to findings from other observational studies, the safety profile of etanercept is favorable also for long-term treatments (Tripathy et al., 2004; Senabre-Gallego et al., 2013). Data from two etanercept pivotal trials revealed no differences in the number of AEs and infections in the etanercept and placebo groups. AEs related to etanercept were injection-site reactions (including swelling), infections (without intergroup differences), and lymphocytopenia (Weinblatt et al., 1999; Blom et al., 2009).

### Serious AEs

As we reported, among 1311 AEs (occurred in 320 patients), 140 were serious. In our opinion the occurrence of such AEs is not surprising for two main reasons. First of all, all serious AEs occurred during the therapy with mAbs. It is well known that biotech drugs, particularly mAbs, can be frequently linked to serious AEs, including infusion reactions, infections, and autoimmune disorders (Tovey and Lallemand, 2011). Indeed, compared to other biotech drugs, mAbs have longer terminal half-lives and, consequently, a single dose of such drugs could lead to prolonged systemic exposure, with an increased risk of serious AEs. Moreover, chimeric mAbs, such as, brentuximab, rituximab, and cetuximab, are more frequently related to serious AEs. For such reason, their use is restricted to the treatment of clinical conditions with high morbidity and mortality (Tranter et al., 2013). Secondly, since traditional RCTs are designed with the aim to avoid risks to enrolled patients, the identification of serious AEs during RCTs is not a simple task. However, when new drugs become available for the use in clinical practice, they are commonly administered to patients not fully represented in RCTs and such patients are the ones more likely to experience serious AEs (Garcia-Doval et al., 2012). Therefore, since in “real life” patients, disease-related risks, comorbidities and concomitant immunosuppressant and chemotherapeutic agents can additionally contribute to the occurrence of AEs (Meadows and Hurwitz, 2012; Kotaka et al., 2016) and considering that almost 40% of our patients had at least one comorbidity, in our opinion, the occurrence of 140 serious AEs should not be considered an alarming figure, rather the consequence of the use of biotech drugs in a widely varied population with characteristics quite different to those of patients enrolled in the traditional RCTs. Nevertheless, scientific evidence is still controversial about risks related to biotech drugs, especially with regard to infections and malignancies risks. Thus, it is important to continue to closely monitor the use of these in clinical practice to improve the knowledge on their long -term safety.

### Treatment discontinuation

Literature data suggest that treatment discontinuation with anti-TNF drugs occur approximately in 21–35% of patients (Combe et al., 2006). According to our findings, results of an internet-based survey by Bolge et al. revealed that lack of effectiveness was the primary reason for discontinuation in rheumatoid arthritis patients receiving etanercept, adalimumab, certolizumab, or golimumab, followed by other AEs occurrence (Bolge et al., 2015). Further clinical data confirm these findings (Weinblatt et al., 2011; Levin et al., 2014; Fafá et al., 2015; Nüßlein et al., 2015).

When biotech drugs are used in oncology setting, their combination with standard chemotherapy increase the risk of AE occurrence leading to more dose reductions or discontinuations (Oza et al., 2017). With this regard, a recent prospective observational cohort study, which enrolled 40 metastatic colorectal cancer patients treated with XELOX and bevacizumab, revealed that among all discontinuations 15 were related to disease progression and 7 to AEs occurrence (Burmester et al., 2013). Also the results of a recent multinational prospective single-arm study revealed that, among 1,021 ovarian cancer patients receiving bevacizumab and paclitaxel, discontinuation occurred in 33% of patients due to disease progression and in 17% of patients due to AEs (da Silva et al., 2014).

## Study limitation

We did not perform a sound statistical analysis for confounders that may have influenced AEs occurrence and we did not consider some important concomitant factors such as, concomitant drug therapies, comorbidities, diseases stage and disease-related risk. Our findings regarding the safety profile of biotech drugs have therefore to be considered exploratory: the small sample size limited our power to detect differences among treatments. Furthermore, the absence of a sample size calculation could have affected the value of our study. Finally, due to the limited follow up period we were not able to detect AEs emerging for long-term treatments, such as, cancer.

## Conclusion

Our study represents one of the activities performed in Campania Region in the field of pharmacovigilance (Rafaniello et al., 2016a; Sessa et al., 2016a,b; Sportiello et al., 2016a,b) with the aim to better define the safety profile of drugs and to improve their safe use in routine clinical practice. The results of our study demonstrated that biotech drugs used in several clinical settings in Campania Region showed overall good tolerability profiles. The majority of identified AEs were not serious and, according to each biotech drug pivotal clinical trial, expected for the respective drugs. Few cases of serious infections were identified, while no case of malignancy occurred. Therefore, no new safety issues emerged from our study.

Nevertheless, some safety concerns still remain unresolved, especially those related to the long-term treatment. In this context, the collection of RWD could add more information on both effectiveness and safety profiles of biotech drugs.

Finally, considering the important role of ADAs on efficacy/safety profile of biotech drugs, in the near future more attention have to be paid to the management of studies based on therapeutic drug monitoring with the aim to evaluate the link between the biotech drug/ADAs concentrations with clinical outcome, including those related to therapeutic failure and infusion/hypersensitivity reactions.

## Author contributions

Drafting the work and revising it for important intellectual content: CS, LS, MGS, CF, RR, MS, PB, Gd, LB, FR, CR, and AC. Substantial contributions to the acquisition, analysis, or interpretation of data for the work: CS, LS, MGS, CF, RR, MS, PMB, Gd, LB, FR, CR, and AC. Final approval of the version to be published: CS, LS, MGS, CF, RR, MS, PB, Gd, LB, FR, CR, and AC. Agreement to be accountable for all aspects of the work in ensuring that questions related to the accuracy or integrity of any part of the work are appropriately investigated and resolved: CS, LS, MGS, CF, RR, MS, PB, Gd, LB, FR, CR, and AC. Developed the concept and designed the study: LB, FR, and AC. Wrote the paper: CS, LS, CR, and AC.

### Conflict of interest statement

The authors declare that the research was conducted in the absence of any commercial or financial relationships that could be construed as a potential conflict of interest.

## Footnotes

^1^I Numeri del Cancro in Italia (2016). Available online at: http://www.registri-tumori.it/PDF/AIOM2016/I_numeri_del_cancro_2016.pdf. (Accessed July 8, 2017)

^2^Malattie reuMatiche: Primo rePort Sull'incidenza delle Esenzioni Per Malattia. Available online at: http://www.quotidianosanita.it/allegati/allegato2570097.pdf (Accessed July 8, 2017).

^3^Humira: EPAR - Product Information. Available online at http://www.ema.europa.eu/docs/en_GB/document_library/EPAR_-_Product_Information/human/000481/WC500050870.pdf.

^4^Remicade: EPAR—Product Information. Available online at http://www.ema.europa.eu/docs/en_GB/document_library/EPAR_-_Product_Information/human/000240/WC500050888.pdf.

^5^Avastin: EPAR - *Product Information*. Available online at http://www.ema.europa.eu/docs/en_GB/document_library/EPAR_-_Product_Information/human/000582/WC500029271.pdf.
