# Supplementary material for: Safety Profile of Anticancer and Immune-Modulating Biotech Drugs Used in a Real World Setting in Campania Region (Italy): BIO-Cam Observational Study
Source: Front Pharmacol. 2017 Sep 6;8:607. doi: 10.3389/fphar.2017.00607 (PMC5592230; doi:10.3389/fphar.2017.00607)
Supplement: Supplementary file 1 [file Table1.DOCX]

Supplementary Material

**Safety Profile of Biotech Drugs Used in a Real World Setting in Campania Region (Italy): BIO-Cam Observational Study**

Cristina Scavone^1^* & Liberata Sportiello^1^*, Maria Giuseppa Sullo^1^, Carmen Ferrajolo^1^, Rosanna Ruggiero^1^, Maurizio Sessa^1^, Pasquale Maria Berrino^1^, Gabriella di Mauro^1^, Liberato Berrino^1^, Francesco Rossi^1^, Concetta Rafaniello^1#^ & Annalisa Capuano^1#^; BIO-Cam Group

^1^ Department of Experimental Medicine – Section of Pharmacology “L. Donatelli” – University of Campania “Luigi Vanvitelli” – Via Costantinopoli, 16, 80138 - Naples (IT), Italy

*these authors have equally contributed

**^#^** these authors are both lead authors

**Corresponding author:**

Cristina Scavone

Department of Experimental Medicine – Section of Pharmacology “L. Donatelli”

University of Campania “Luigi Vanvitelli” – Naples (IT), Italy

Email: [cristina.scavone@unicampania.it](mailto:cristina.scavone@unicampania.it)

Tel: 00390815665805

Fax: 00390815667652

**Supplementary Table 1.** **Distribution of Adverse Event in terms of System Organ Class(SOC) and time of follow-up.**

|  | AE at time of follow-up | | | | | | | | | | | | | | | |
| --- | --- | --- | --- | --- | --- | --- | --- | --- | --- | --- | --- | --- | --- | --- | --- | --- |
| SOC | at injection | | from 1 to 30 days | | from 31-60 days | | from 61 to 90 days | | from 91 to 180 days | | from 181 to 360 days | | >361 days | | All AEs | |
|  | N | (%) | N | (%) | N | (%) | N | (%) | N | (%) | N | (%) | N | (%) | N | (%) |
| **tot** | **157** | **12.0%** | **222** | **16.9%** | **227** | **17.3%** | **168** | **12.8%** | **277** | **21.1%** | **216** | **16.5%** | **44** | **3.4%** | **1311** | **100.0%** |
| Gastrointestinal disorders | 20 | 8.3% | 43 | 17.8% | 50 | 20.7% | 32 | 13.2% | 40 | 16.5% | 46 | 19.0% | 11 | 4.5% | 242 | 100.0% |
| Skin and subcutaneous tissue disorders | 34 | 16.6% | 45 | 22.0% | 32 | 15.6% | 22 | 10.7% | 40 | 19.5% | 26 | 12.7% | 6 | 2.9% | 205 | 100.0% |
| Nervous system disorders | 16 | 8.1% | 21 | 10.6% | 27 | 13.6% | 26 | 13.1% | 55 | 27.8% | 47 | 23.7% | 6 | 3.0% | 198 | 100.0% |
| General disorders and administration site conditions | 19 | 9.9% | 32 | 16.7% | 36 | 18.8% | 23 | 12.0% | 41 | 21.4% | 32 | 16.7% | 9 | 4.7% | 192 | 100.0% |
| Blood and lymphatic system disorders | 11 | 13.8% | 16 | 20.0% | 15 | 18.8% | 12 | 15.0% | 19 | 23.8% | 7 | 8.8% |  |  | 80 | 100.0% |
| Musculoskeletal and connective tissue disorders | 5 | 7.1% | 6 | 8.6% | 15 | 21.4% | 12 | 17.1% | 20 | 28.6% | 9 | 12.9% | 3 | 4.3% | 70 | 100.0% |
| Vascular disorders | 15 | 22.4% | 15 | 22.4% | 9 | 13.4% | 10 | 14.9% | 8 | 11.9% | 9 | 13.4% | 1 | 1.5% | 67 | 100.0% |
| Respiratory, thoracic and mediastinal disorders | 16 | 23.9% | 17 | 25.4% | 9 | 13.4% | 7 | 10.4% | 6 | 9.0% | 9 | 13.4% | 3 | 4.5% | 67 | 100.0% |
| Infections and infestations | 2 | 4.7% | 8 | 18.6% | 10 | 23.3% | 6 | 14.0% | 12 | 27.9% | 3 | 7.0% | 2 | 4.7% | 43 | 100.0% |
| Eye disorders | 2 | 7.4% | 3 | 11.1% | 6 | 22.2% | 3 | 11.1% | 8 | 29.6% | 5 | 18.5% |  |  | 27 | 100.0% |
| OTHER SOC* | 17 | 14.2% | 16 | 13.3% | 18 | 15.0% | 15 | 12.5% | 28 | 23.3% | 23 | 19.2% | 3 | 2.5% | 120 | 100.0% |
