# Supplementary material for: Safety Profile of Anticancer and Immune-Modulating Biotech Drugs Used in a Real World Setting in Campania Region (Italy): BIO-Cam Observational Study
Source: Front Pharmacol. 2017 Sep 6;8:607. doi: 10.3389/fphar.2017.00607 (PMC5592230; doi:10.3389/fphar.2017.00607)
Supplement: Supplementary file 2 [file Table2.DOCX]

Supplementary Material

**Safety Profile of Biotech Drugs Used in a Real World Setting in Campania Region (Italy): BIO-Cam Observational Study**

Cristina Scavone^1^* & Liberata Sportiello^1^*, Maria Giuseppa Sullo^1^, Carmen Ferrajolo^1^, Rosanna Ruggiero^1^, Maurizio Sessa^1^, Pasquale Maria Berrino^1^, Gabriella di Mauro^1^, Liberato Berrino^1^, Francesco Rossi^1^, Concetta Rafaniello^1#^ & Annalisa Capuano^1#^; BIO-Cam Group

^1^ Department of Experimental Medicine – Section of Pharmacology “L. Donatelli” – University of Campania “Luigi Vanvitelli” – Via Costantinopoli, 16, 80138 - Naples (IT), Italy

*these authors have equally contributed

**^#^** these authors are both lead authors

**Corresponding author:**

Cristina Scavone

Department of Experimental Medicine – Section of Pharmacology “L. Donatelli”

University of Campania “Luigi Vanvitelli” – Naples (IT), Italy

Email: [cristina.scavone@unicampania.it](mailto:cristina.scavone@unicampania.it)

Tel: 00390815665805

Fax: 00390815667652

**Table 2. Description of cases of therapy discontinuation by biologic/biotech drug.**

|  | **Cases of discontinuation N.** | **Reason of discontinuation** |
| --- | --- | --- |
| **abatacept** | 6 | AE (2); systemic vasculitis; stroke |
| **adalimumab** | **2** | Lymphopenia (1); DTF*(2) |
| **bevacizumab** | 7 | DTF*(4); progression disease (3) |
| **certolizumab pegol** | 3 | AE (1); DTF*(2); low compliance (1) |
| **cetuximab** | 3 | AE (1); progression disease (1); DTF*(1) |
| **denosumab** | 3 | Death^$^ (3) |
| **erlotinib** | 4 | Death^$^ (2), progression disease (2) |
| **etanercept** | 7 | DTF*(4); AE (3) |
| **golimumab** | 2 | DTF*(1); AE (1) |
| **infliximab** | 6 | DTF*(4); Change of address (1); AE (1) |
| **panitumumab** | 1 | progression disease (1) |
| **rituximab** | 1 | DTF*(1) |
| **tocilizumab** | 3 | Lymphopenia (1); AE (1); low compliance (1) |
| **trastuzumab** | 2 | End of therapy (1); DTF*(1) |

*DTF: Drug therapeutic failure

^$^The deaths are not induced by biotech drugs but represent the reason of discontinuation of the pharmacological treatment.
